# Composition Regulation of Potassium Sodium Niobate Thin Films through Post-Annealing under Alkali Element Atmospheres

**DOI:** 10.3390/nano14030288

**Published:** 2024-01-30

**Authors:** Binjie Chen, Chuanyang Tao, Wenying Fan, Binglin Shen, Min Ju, Zhongshang Dou, Chaofeng Wu, Fang-Zhou Yao, Wen Gong, Ke Wang

**Affiliations:** 1 Research Center for Advanced Functional Ceramics, Wuzhen Laboratory, Jiaxing 314500, China; 2 Tongxiang Tsingfeng Technology Co., Ltd., Jiaxing 314501, China; 3 Center of Advanced Ceramic Materials and Devices, Yangtze Delta Region Institute of Tsinghua University, Jiaxing 314006, China; 4 State Key Laboratory of New Ceramics and Fine Processing, School of Materials Science and Engineering, Tsinghua University, Beijing 100084, China

**Keywords:** potassium sodium niobate, piezoelectric, RF sputtering, post-annealing

## Abstract

Amorphous potassium sodium niobate (KNN) films were synthesized at 300 °C through the radio frequency magnetron sputtering method and subsequently crystallized by post-annealing at 700 °C in various alkali element atmospheres (Na and K). The as-deposited film is notably deficient in alkali metal elements, particularly K, whereas the loss of alkali elements in the films can be replenished through annealing in an alkali element atmosphere. By adjusting the molar ratio of Na and K in the annealing atmosphere, the ratio of Na/K in the resultant film varied, consequently suggesting the efficiency of this method on composition regulation of KNN films. Meanwhile, we also found that the physical characteristics of the films also underwent differences with the change of an annealing atmosphere. The films annealed in a high Na atmosphere exhibit large dielectric losses with limited piezoelectric vibration behavior, while annealing in a high K atmosphere reduces the dielectric losses and enhances the piezoelectric behavior. Furthermore, the results of vibration measurement demonstrated that the film annealed in a mixed powder of 25% Na_2_CO_3_ and 75% K_2_CO_3_ exhibits an optimal vibration displacement of ~400 pm under the sinusoidal excitation voltage of 8 V. This approach of altering the composition of KNN films through post-annealing may introduce the new concept of property design of KNN as well as other similar films.

## 1. Introduction

As heightened concerns regarding environmental issues associated with lead (Pb) continue to grow, the substitution of lead-based piezoelectric materials with lead-free alternatives has garnered significant attention [1,2]. Among various materials, potassium sodium niobate (KNN) has been extensively studied owing to its promising piezoelectric-related characteristics, such as large piezoelectric coefficients comparable to that of lead-based ceramics, high Curie temperature, and high modifiability through composition regulation and texture design [3,4,5,6,7]. Currently, notable progress has been achieved in employing KNN ceramic bulk across various applications such as transducers, sensors, etc. [8,9,10]. Meanwhile, some non-traditional applications based on KNN ceramics, such as piezoelectric composite fillers in flexible materials, have also been proposed [11,12]. However, with the continuous development of micro-nano technology, demand for miniaturization of piezoelectric devices is also growing, giving rise to the requirement for piezoelectric materials to be thin film and integrated with silicon-based semiconductor processes [13,14,15]. Therefore, realizing the synthesis of high-quality KNN films represents both a major challenge and a significant opportunity to advance the industrialization of lead-free piezoelectrics.

Up to now, KNN and KNN-based films have been reported to be fabricated on different substrates using various methods, including pulsed laser deposition (PLD), sol-gel method, radio frequency (RF) magnetron sputtering, etc. [16,17,18]. However, consistent with the issue encountered in the preparation of KNN ceramic bulks, the films almost inevitably suffer from non-stoichiometric problems due to the thermal evaporation of alkali elements of potassium and sodium [19], which eventually results in the property deterioration of KNN films. In particular, during physical vapor deposition processes such as RF magnetron sputtering and PLD, the plasma ionization further exacerbates the volatility of alkali elements, making it even more difficult to effectively control the growth of KNN films [20,21]. Actually, previous studies have already revealed that the sputtering growth of KNN films typically employs the target with large alkali metal element excess [20,21,22,23,24]. Wang et al. used a low-density target with the molar ratio of K:Na:Nb = 1.5:1.5:1 to grow KNN films on LaAlO_3_ substrates by RF magnetron sputtering, successfully fabricating the stoichiometric films [21]. Based on the target with the same molar ratio, Lee et al. have also continued to explore sputtering parameters for the growth of KNN films [22,25,26]. It is suggested that alkali metal deficiency is unavoidable when targets with stoichiometric ratios are used as sputtering sources. However, there are still some reports of sputtered films produced from stoichiometric targets and then adjusting the composition and properties through subsequent post-annealing processes [27,28]. For instance, Kim et al. annealed the films in K_2_O, Na_2_O, and KNN atmosphere, revealing that the Na-excess film, K-excess film, and homogeneous film with a composition close to (K_0.5_Na_0.5_)NbO_3_ were formed, respectively [27]. However, for one thing, the origin of the atmosphere was not clearly elucidated in their study. And for another, several secondary phases were also observed, probably due to improper annealing parameters. It is evident that the atmospheric post-annealing process effectively compensates for the alkali element for KNN films. Nevertheless, there remains an absence of comprehensive studies on the regular modulation of the composition of KNN film through such a method.

In this study, potassium carbonate (K_2_CO_3_) and sodium carbonate (Na_2_CO_3_) powders were employed to generate the alkali element atmosphere during the post-annealing process of KNN films. Changes in the composition and physical properties of annealed films under various atmospheres with different Na/K ratios were systematically investigated. As a result, pure polycrystal KNN films were obtained and exhibited a distinct dependence on the annealing atmosphere in terms of composition and physical characteristics. Notably, the film annealed in a mixed powder of 25% Na_2_CO_3_ and 75% K_2_CO_3_ exhibits optimal ferroelectric and piezoelectric properties among all resultant films.

## 2. Materials and Methods

The KNN films were fabricated on commercial Pt (150 nm)/Ti (20 nm)/SiO_2_ (300 nm)/Si substrates by radio frequency (RF) magnetron sputtering. A stoichiometric KNN ceramic (K: Na = 1:1) synthesized with 1 wt% MnO_2_ as sintering additive via solid-state reaction method was used as the sputtering target. The deposition of KNN films was performed at the substrate temperature (*T*_sub_) of 300 °C in a mixed Ar/O_2_ (4:1) atmosphere with total pressure of 1.3 Pa. The thickness of all films was controlled to ~250 nm by unifying the sputtering power (120 W) and duration (2 h). After the film deposition, the films were further post-annealed at 700 °C for 2 h in various atmospheres of alkali elements, which were produced from Na_2_CO_3_ and K_2_CO_3_ powder mixed with different molar ratios. To obtain the dielectric properties and vibration information of the resultant films, a circular Pt electrode with 100 μm diameter as top electrode was deposited by sputtering with shadow mask.

The crystal structure of the KNN films was determined using grazing incidence X-ray diffraction (GIXRD, D8 Advance, Bruker, Billerica, MA, USA) with incidence angle of 1°. The film’s chemical composition and distribution were analyzed using scanning electron microscopy/energy dispersive spectrometry (SEM-EDS, Sigma 300, Zeiss, Oberkochen, Germany). The morphology and ferroelectric information were analyzed through piezoresponse force microscopy (PFM, Jupiter XR, Oxford Instruments, Abingdon, UK). Dual AC Resonance Tracking (DART) PFM was first used to observe the morphology and intrinsic domain structure of the resultant films prior to applying ferroelectric inversion. Subsequently, PFM Lithography was employed for domain writing to achieve the localized domain inversion. Finally, DART-PFM was performed again to characterize the domain inversion information and demonstrate the ferroelectricity of the films. The frequency dependence of dielectric properties was measured using precision impedance analyzer (E4990A, Keysight, Santa Rosa, CA, USA). The piezoelectric vibration was generated by applying sinusoidal excitation voltage and monitored using a microscope-based laser Doppler vibrometer (LDV, MSA-100-3DSV, Polytec, Waldbronn, Germany).

## 3. Results and Discussion

**Figure 1:**
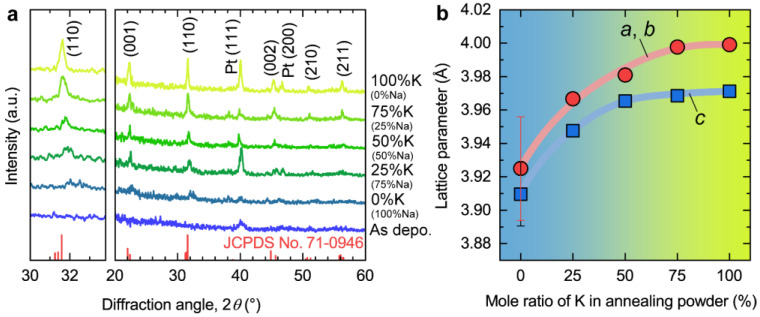
Diffraction characterization. (**a**) Grazing incidence X-ray diffraction (GIXRD) patterns of the resultant KNN films grown on Pt/Ti/SiO_2_/Si substrates. The different color of the lines reprsent the data for films annealed under various atmospheres. The red vertical lines below indicate the XRD pattern of JCPDS No. 71-0946. (**b**) The lattice parameters of the resultant KNN films are the function of the molar ratio of K in annealing powder. The data were extracted from the GIXRD patterns, and it was assumed that the crystal structure is an equivalent tetragonal structure, *a* ≈ *b* ≠ *c*.

Figure 1a shows the GIXRD patterns of the as-deposited and annealed KNN films grown on Pt/Ti/SiO_2_/Si substrates. For the as-deposited film, no other peaks except for the diffraction peak of Pt (111) at 2 *θ* = ~40° can be observed, indicating its amorphous nature. In contrast, the films that underwent post-annealing at 700 °C exhibit substantial diffraction information: (1) The typical diffraction peaks at 2 *θ* = ~22.5°, 31.6°, 45.4°, 50.8°, and 56.5° can be well indexed based on the JCPDS No. 71-0946 and attributed to the crystal planes of (001), (110), (002), (210), and (211) of the KNN crystal, respectively, which confirms that all the annealed films are polycrystalline. (2) The overall peak intensity of annealed films gradually increases with the increasing molar proportion of potassium (K) in the mixed powder used for annealing. This suggests that the film crystallizes more readily in a K-rich atmosphere. (3) Also, with the increasing molar proportion of K, the diffraction peaks of KNN (001) and KNN (110) shifted to small diffraction angles simultaneously, suggesting the enlargement of the cell size of KNN. To clearly show the change trends of cell size, we extracted the lattice parameters based on the diffraction peaks of KNN (001) and KNN (110) of GIXRD patterns (assuming the crystal structure is an equivalent tetragonal structure, *a* ≈ *b* ≠ *c*) and plotted them as the function of the molar ratio of K in annealing powder, as shown in Figure 1b. Both lattice parameters *a* (or *b*) and *c* gradually increase from ~3.925 Å to ~3.999 Å and from ~3.910 Å to 3.971 Å, respectively, as the molar ratio of K in annealing powder increases. It is well consistent with the change tendency of lattice parameter as the function of K/(K + Na) composition of the KNN ceramics [29]. Accordingly, we hypothesized that the composition of the resultant KNN film synthesized in this study also underwent a regularized change during the post-annealing process.

**Figure 2:**
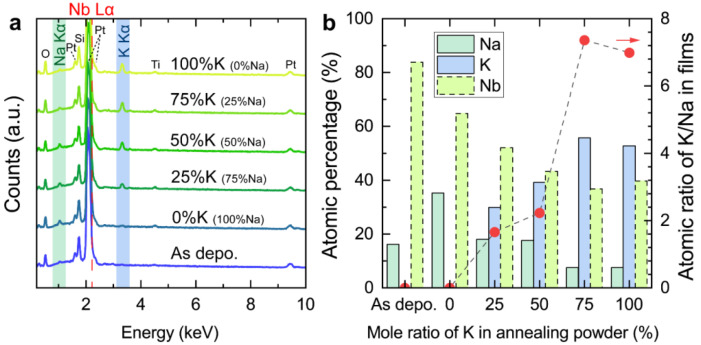
Composition analysis. (**a**) The SEM-EDS spectra of the resultant KNN films. The different color of the lines reprsent the data for films annealed under various atmospheres. The red dashed line represents the standard energy of Nb Lα, which overlaps with the peak of Pt Mα. (**b**) The atomic percentage of Na, K, and Nb and atomic ratio of K/Na as the function of the molar ratio of K in annealing powder. The border of Nb was marked as dashed lines due to the potential inaccuracies in its quantified values.

To intuitively visualize the composition regulation of KNN film in this study, SEM-EDS analyses were performed for the as-deposited film as well as all annealed films. On the one hand, EDS mapping results indicate all elements in the films exhibited highly homogeneous distribution (Figure S1, EDS mapping of KNN film annealed at 75% K_2_CO_3_ and 25% Na_2_CO_3_ powder as an example). On the other hand, the characteristic peaks of elements in the EDS spectra exhibited systematic variations, as shown in Figure 2a. The peaks at ~0.53, ~1.60, ~1.74, ~2.06, ~2.33, ~4.52, and ~9.42 keV can be assigned to O Kα, Pt M*, Si Kα, Pt Mα, Pt Mγ, Ti Kα, and Pt Lα, respectively. Their peak intensity did not change significantly with annealing atmosphere changes, suggesting that their origin is possibly not exclusively from the film but from the substrate. On the contrary, the peaks at ~1.05 and ~3.31 keV, attributed to Na Kα and K Kα, respectively, show significant regularity trends. For the as-deposited film, a weak peak of Na Kα was observed solely without the appearance of K Kα, implying the strong alkali element volatilization during sputtering. When annealing the films in atmospheres of alkali elements, the Na Kα peak first increased when the pure Na_2_CO_3_ powder was used and subsequently decreased as the proportion of K_2_CO_3_ in mixed powder grew, while the K Kα peak gradually increased. Both indicate that the alkali element enters the interior of the film during the annealing process to compensate for the original deficiency of the as-deposited film. To quantify the above phenomenon, we summarized the relative atomic percentage of Na, K, and Nb elements, as shown in Figure 2b. It is necessary to note that the characteristic peak of Nb overlaps with that of Pt, which may result in the inaccurate quantized values of Nb. The quantitative change trends of Na and K correspond exactly to the results of EDS spectra. Moreover, the K in the film increases rapidly with the change of annealing powder composition and overtakes Na as the major element in the A-site of KNN. The above results suggest that atmospheric post-annealing is an effective method to achieve alkali element replenishment in KNN films, and the K replenishment was superior to that of Na.

**Figure 3:**
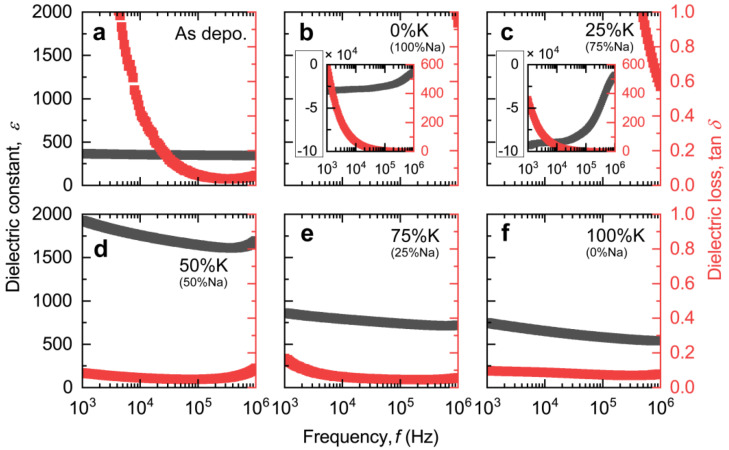
Dielectric properties of the resultant films. Dielectric constant (*ε*) and loss tangent (tan *δ*) of (**a**) as-deposited film, (**b**) the film annealed in pure Na_2_CO_3_ powder, (**c**) the film annealed in mixed powder of 75% Na_2_CO_3_ and 25% K_2_CO_3_, (**d**) the film annealed in mixed powder of 50% Na_2_CO_3_ and 50% K_2_CO_3_, (**e**) the film annealed in mixed powder of 25% Na_2_CO_3_ and 75% K_2_CO_3_, and (**f**) the film annealed in pure K_2_CO_3_ powder as the function of frequency (1 kHz~1 MHz). The insets in (**b**,**c**) are the axis-rescaled images to show all values. Note that the *ε* of (**b**) the film annealed in pure Na_2_CO_3_ powder and (**c**) the film annealed in mixed powder of 75% Na_2_CO_3_ and 25% K_2_CO_3_ are negative values due to the high tan *δ*.

For KNN films, it has been reported that the absence of K and Na will severely affect the electrical properties and microstructure. Figure 3 shows the dielectric properties (dielectric constant (*ε*) and loss tangent (tan *δ*)) of the resultant KNN films as the function of frequency (1 kHz~1 MHz). The *ε* of the as-deposited film (Figure 3a) is ~350, demonstrating nominal frequency dependency. However, when annealing the film in an atmosphere with a high proportion of Na (Figure 3b,c), a negative *ε*, instead of a positive *ε* usually obtained in conventional dielectric materials, was observed and accompanied by an enormous dielectric loss in these two films. Such a phenomenon is usually due to inductive behavior resulting from excessive leakage current. More intensively, it is closely associated with the crystallization processes of KNN film during the annealing process. The as-deposited films (amorphous) typically exhibit relatively lower leakage currents and smaller tan *δ* due to their lack of long-range ordering, which limits their internal charge transport, whereas the film after annealing (polycrystalline) possesses a certain structural ordering, accompanied by the formation of conductive pathways due to the absence of K and Na. The combination of the above two factors eventually leads to a huge leakage of polycrystal films. Accordingly, when annealing the as-deposited film in an atmosphere of higher K concentration (Figure 3d–f), the loss of alkali elements in the A-site is effectively compensated, as we have previously observed in EDS spectra. Thus, the tan *δ* of these three films are 0.08, 0.15, and 0.09@1 kHz and 0.11, 0.05, and 0.08@1 MHz, respectively, much lower than those of the films annealing in an atmosphere of higher K concentration (Figure 3b,c). In addition, we also noticed that the overall *ε* of these three films also decreases with increasing K concentration in the atmosphere, which is consistent with the reported trend of decreasing *ε* in KNN films as the molar ratio of K increases [30].

**Figure 4:**
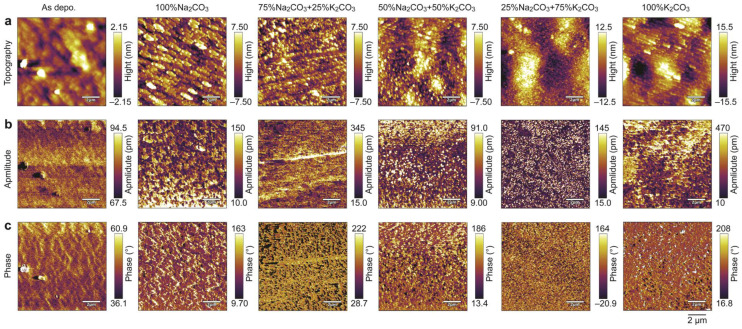
PFM images of the resultant films captured at a scale of 10 × 10 μm. (**a**) Topography images. (**b**) Amplitude images. (**c**) Phase images. The scale bar for all images is 2 μm.

**Figure 5:**
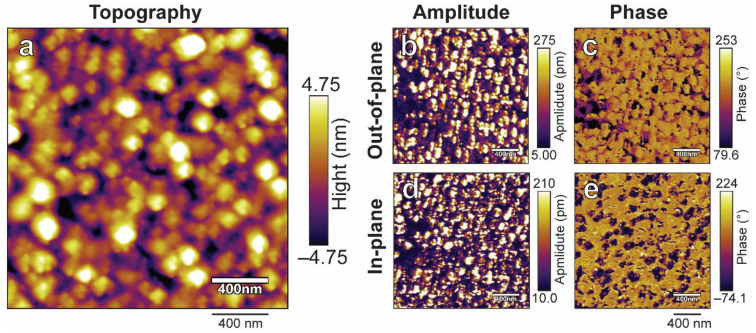
Enlarged PFM images of the film annealed in mixed powder of 25% Na_2_CO_3_ and 75% K_2_CO_3_ captured at a scale of 2 × 2 μm. (**a**) Topography image. (**b**,**c**) Out-of-plane (**b**) amplitude and (**c**) phase images. (**d**,**e**) In-plane (**d**) amplitude and (**e**) phase images. The scale bar for all images is 400 nm.

**Figure 6:**
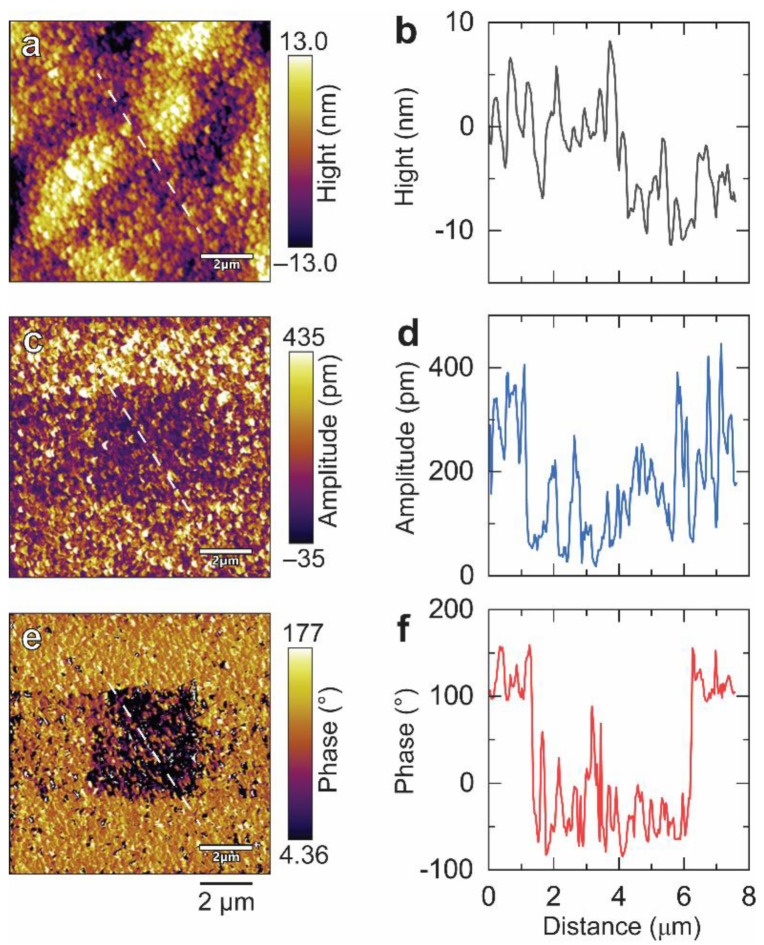
PFM images of the film annealed in mixed powder of 25% Na_2_CO_3_ and 75% K_2_CO_3_ after applying switching bias of ±10 V. (**a**) Topography image. (**b**) The corresponding line scan of topography. (**c**) Amplitude image. (**d**) The corresponding line scan of amplitude. (**e**) Phase image. (**f**) The corresponding line scan of phase. The scale bar in (**a**–**c**) is 2 μm. The white dashed lines shown in (**a**,**c**,**e**) indicate the route of line scan.

The microscopic information of all resultant films was also characterized by PFM techniques, as depicted in Figure 4. The root-mean-square (RMS) roughness of topography gradually increases from 1.4 nm of as-deposited film to 9.5 nm of the film annealed in 100% K_2_CO_3_, corresponding to the enhancement of the crystallinity of the films as shown in GIXRD (Figure 1a). The amplitude image and phase image can be used in combination to evaluate domain and polarization information. Intuitive amplitude and phase contrasts that do not arise exclusively from topography were observed in all samples except the as-deposited film, suggesting that the annealed film possesses some spontaneous polarization. It is worth noting that the overall domain size varies with the annealing atmosphere and the film annealed in mixed powder of 25% Na_2_CO_3_ and 75% K_2_CO_3_ possesses the smallest domain size by visual comparison. We also conducted more detailed observations on it. Figure 5 shows the PFM images of this film within the plane scale of 2 × 2 μm. The topography image (Figure 5a) reveals that the film has a nanosized grain-feature structure with a grain size of 100~200 nm. Both out-of-plane and in-plane images of amplitude (Figure 5b,d) and phase (Figure 5c,e) show that the film possesses a mono-grain-like domain structure rather than the strip-like domain typically observed in KNN ceramic bulk. Such a grain domain structure has also been observed in previous reports of KNN films [31,32], likely attributed to the insufficient size of the nanoscale grains, which are unable to accommodate larger strip domains. Furthermore, phase-field simulations indicated that within the nanoscale, smaller domains imply that less energy is needed to achieve ferroelectric inversion [33], and it has also been preliminarily noted in KNN-based materials [34]. To clarify the ferroelectric inversion characteristics, the opposite bias of ±10 V was applied to the center and surrounding area using a conductive tip. Subsequently, the PFM amplitude and phase signals were recorded once more. Figure 6 shows the obtained PFM topography, amplitude, phase image, and the corresponding line scan through the biased area. The topography of biased films slightly changed (Figure 6a), possibly due to the change of charge state on the film surface or the change of resonance of tip [35,36], while the domain changes much more significantly compared to that of topography. It can be clearly observed that the domains of the film were reversed. The domain in the center area underwent a downward switching when −10 V was applied to this area, while the domain in the surrounding area was switched upwards after poling with +10 V bias (Figure 6e). In addition to this, the line scan of phase (Figure 6f) further confirms that the phase difference between the center and surrounding area is ~180°. The above results illustrate that the film annealed in the mixed powder of 25% Na_2_CO_3_ and 75% K_2_CO_3_ possesses good ferroelectric inversion behavior. Additionally, we also performed similar measurements on all other KNN films (Figure S2); only this sample exhibited the most significant ferroelectric inversion in the instrumental limit, which is possibly relevant to our extrapolation on the domain size effect.

**Figure 7:**
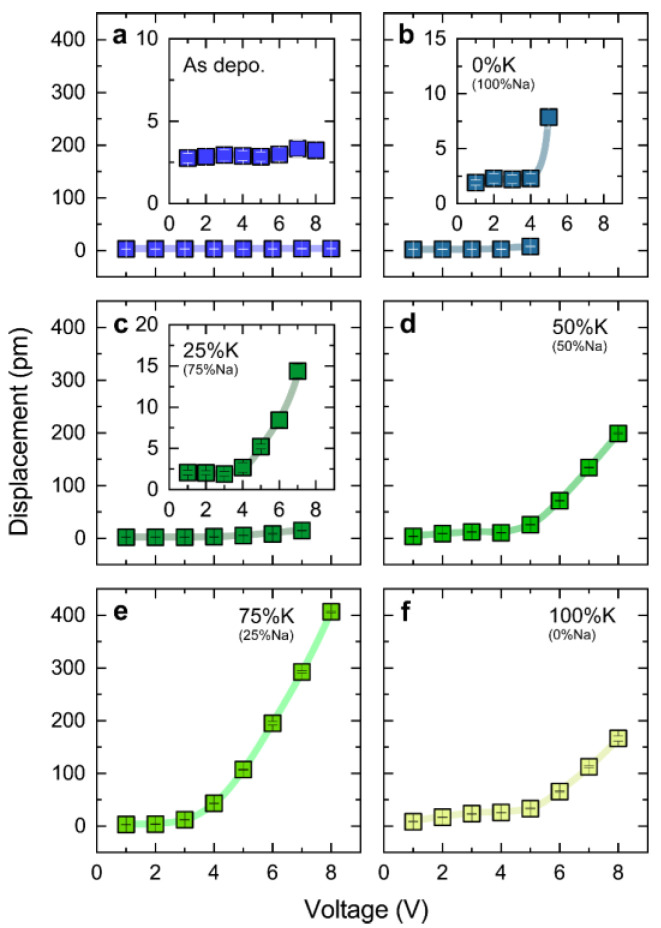
Piezoelectric vibration of the resultant films when applying sinusoidal excitation with the various voltages of 1–8 V. (**a**) As-deposited film. (**b**) The film annealed in pure Na_2_CO_3_ powder. (**c**) The film annealed in mixed powder of 75% Na_2_CO_3_ and 25% K_2_CO_3_. (**d**) The film annealed in mixed powder of 50% Na_2_CO_3_ and 50% K_2_CO_3_. (**e**) The film annealed in mixed powder of 25% Na_2_CO_3_ and 75% K_2_CO_3_. (**f**) The film annealed in pure K_2_CO_3_ powder. The frequency of sinusoidal excitation was fixed to 3 kHz. The insets in (**a**–**c**) are the detailed data displayed within a smaller scale of y-axis for clarity. The error bars were estimated based on the data obtained from five repeated measurements.

To investigate the macroscopic piezoelectric characteristic of the resultant films, we employed a laser Doppler vibrometer to observe the vibration of all films when applying sinusoidal excitation of 3 kHz, where this frequency was adopted due to the frequency-dependent measurement of the film annealed in the mixed powder of 25% Na_2_CO_3_ and 75% K_2_CO_3_ illustrated in Figure S3, which demonstrates that the displacement was found to be more pronounced at lower frequencies. However, frequencies below 3 kHz are susceptible to environmental disturbances. In fact, the displacement shows a strong frequency-dependent feature, decreasing almost linearly with increasing frequency. Such behavior is usually attributed to the additional contribution of domain wall motion. Furthermore, this contribution is also responsible for the nonlinear piezoelectric response versus the applied electric field [37,38]. Figure 6 summarizes the electric field dependence of displacement of all resultant KNN films measured under the AC voltage with a magnitude ranging from 1 to 8 V. The as-deposited film did not show significant displacement even at 8 V (Figure 7a). On the contrary, the annealed films gradually exhibit vibrational behavior with the change in the annealing atmosphere. For the films annealed in the atmosphere with a high proportion of Na (Figure 7b,c), no displacement above the instrumental background was observed when the excitation voltage was below 4 V, whereas displacement appeared and gradually increased with increasing voltage. However, due to the poor insulating properties of the two films themselves, it is not possible to apply an AC voltage up to 8 V. Indeed, this corresponds to the high loss tangent (tan *δ*) of these two films in dielectric measurement (Figure 3). When further increasing the K concentration in the atmosphere, the displacement of the film becomes more distinctive, reaching more than 150 pm at 8 V (Figure 7d–f). We hypothesize that the overall effect of such an increase stems from the sufficient replenishment of alkali elements in the A-site. Meanwhile, it should be noted that the film annealed in the mixed powder of 25% Na_2_CO_3_ and 75% K_2_CO_3_ exhibits optimal displacement characteristics, reaching ~400 pm at 8V (Figure 7e). According to the converse piezoelectric effect, the piezoelectric coefficient (*d*_33_*) can be roughly estimated to be ~50 pm/V [39], which is comparable to that of KNN films prepared using the target with excess alkali metal elements [22]. The remarkable performance is consistent with the results of previous PFM observations, implying a relationship between domain size, ferroelectric inversion, and piezoelectric properties. In fact, enhancement of piezoelectric performance, induced by the downsized domain, has been observed in other piezoelectric ceramics and attributed to the higher domain wall density [40,41,42]. Since the piezoelectric properties strongly depend on the motion of the domain walls under the external field, smaller domains increase the domain wall density, further enhancing the piezoelectric properties [42,43]. Additionally, it is worth noting that the nonlinear behavior is also clearly observed in these three samples, corresponding to the frequency dependence shown in Figure S3. This further implies the critical role of the domain wall contribution in the piezoelectric behavior of the resultant films.

**Figure 8:**
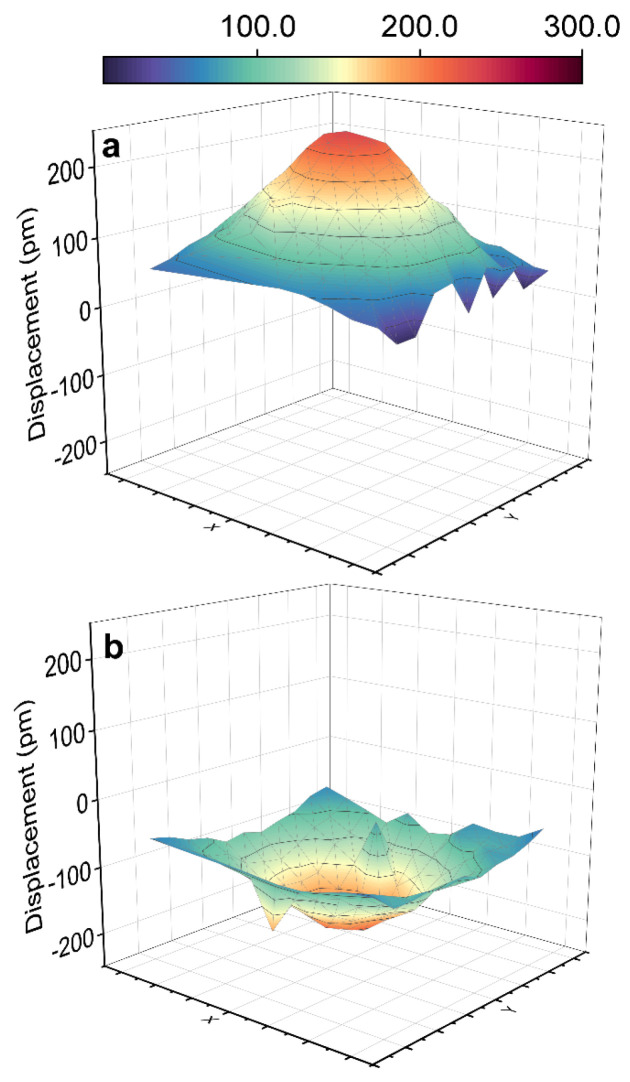
The 2D vibration scan of the film annealed in mixed powder of 25% Na_2_CO_3_ and 75% K_2_CO_3_. (**a**) Upward vibration state. (**b**) Downward vibration state.

To further confirm the dynamized vibration condition of the film to exclude the influence of environmental and static factors, we performed the 2D vibration scan around the circular top electrode, and the results are shown in Figure 8. Here, a sinusoidal excitation with a voltage of 6 V and a frequency of 3 kHz was employed to stimulate vibrational displacements. The transient upward and downward vibration states in the electrode region can be clearly observed, and the maximum displacement is ~200 pm, consistent with the value acquired in a single-point measurement (Figure 7e). On the contrary, no significant displacement occurs outside the electrode region. The above results suggest that the vibrations originate from the intrinsic properties of the film. Thus, we confirm that the films annealed under different alkali element atmospheres exhibit differentiated piezoelectric characteristics.

## 4. Conclusions

In this study, we systematically investigated the effects of post-annealing in alkali element atmospheres with various Na/K ratios on the crystal structure, domain structure, and physical properties of KNN thin films. We found that the alkali metal elements in the films can be selectively replenished and regulated by such an annealing process. As a result, the lattice parameters of the films were distinctly modulated. Meanwhile, significant differences in macroscopic dielectric, piezoelectric properties and microscopic ferroelectric inversion behavior of the resultant films were also observed. Notably, both the most pronounced ferroelectric inversion and the optimal piezoelectric vibrational behavior appear in the film annealed in the mixed powder of 25% Na_2_CO_3_ and 75% K_2_CO_3_, which is probably associated with its smaller ferroelectric domain size. This study introduces a new method to modulate the film composition through post-annealing, presenting a promising path for the property design of KNN films.

## Data Availability

The data that supports the findings of this study are available from the authors upon reasonable request.

## Supplementary Materials

The following supporting information can be downloaded at https://www.mdpi.com/article/10.3390/nano14030288/s1. Figure S1: SEM-EDS mapping of KNN film annealed in mixed powder of 25% Na_2_CO_3_ and 75% K_2_CO_3_.; Figure S2: PFM images of the resultant films after applying switching bias of ±10 V; Figure S3: Frequency dependence of displacement of the film annealed in mixed powder of 25% Na_2_CO_3_ and 75% K_2_CO_3_.
