# Supplementary material for: Composition Regulation of Potassium Sodium Niobate Thin Films through Post-Annealing under Alkali Element Atmospheres
Source: Nanomaterials (Basel). 2024 Jan 30;14(3):288. doi: 10.3390/nano14030288 (PMC10856795; doi:10.3390/nano14030288)
Supplement: Supplementary file 1 [file nanomaterials-14-00288-s001.zip › nanomaterials-2837884-supplementary.pdf]

Supplementary Materials for:

Composition Regulation of Potassium Sodium Niobate Thin Films through Post-Annealing under Alkali Element Atmospheres

Binjie Chen [1,*], Chuanyang Tao [1], Wenying Fan [1], Binglin Shen [1], Min Ju [1], Zhongshang Dou [1], Chaofeng Wu [2], Fang-Zhou Yao [1,3,*], Wen Gong [2,*] and Ke Wang [1,4]

1 Research Center for Advanced Functional Ceramics, Wuzhen Laboratory, Jiaxing 314500, China; tcy4174@163.com (C.T.); fanwy@wuzhenlab.com (W.F.); shenbl@wuzhenlab.com (B.S.); jum@wuzhenlab.com (M.J.); douzs@wuzhenlab.com (Z.D.); wang-ke@tsinghua.edu.cn (K.W.)

2 Tongxiang Tsingfeng Technology Co., Ltd., Jiaxing 314501, China; wucf@mail.tsinghua-zj.edu.cn (C.W.)

3 Center of Advanced Ceramic Materials and Devices, Yangtze Delta Region Institute of Tsinghua University, Jiaxing 314006, China

4 State Key Laboratory of New Ceramics and Fine Processing, School of Materials Science and Engineering, Tsinghua University, Beijing 100084, China

* Correspondence: chenbj@wuzhenlab.com (B.C.); yaofangzhou@xjtu.edu.cn (F.-Z.Y.); pgong@139.com (W.G.)

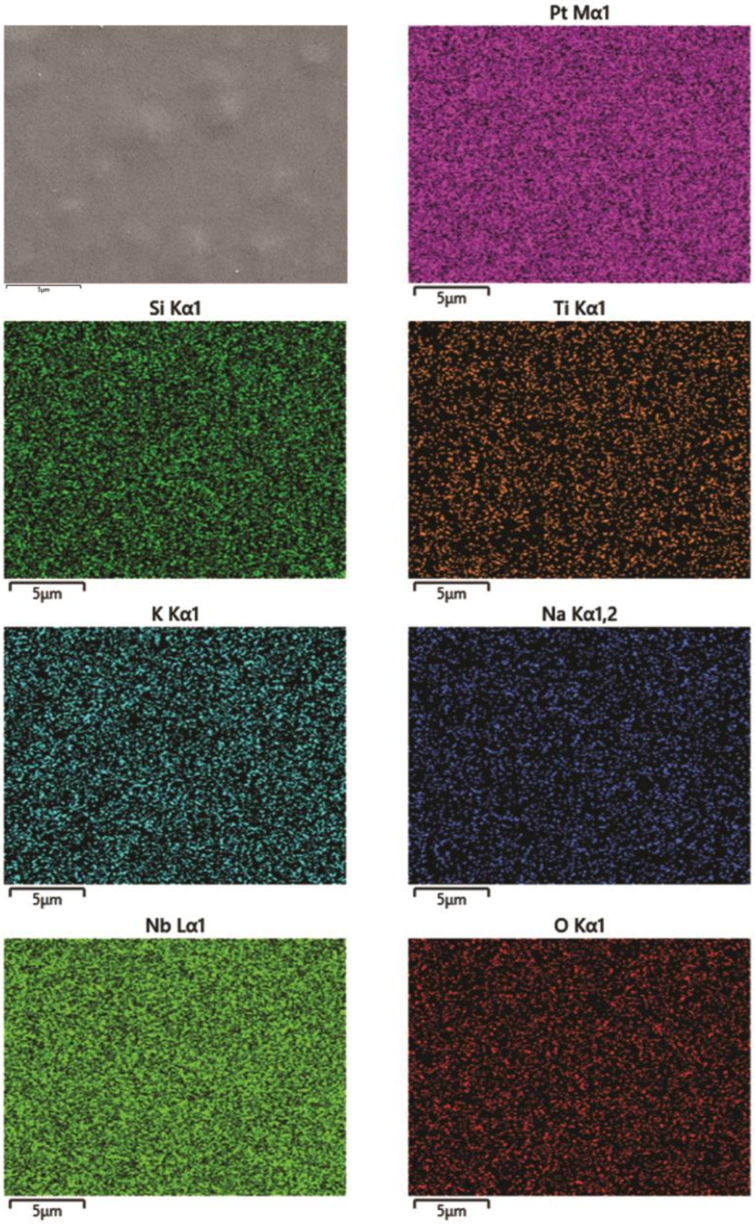

Figure S1. SEM- EDS mapping of KNN film annealed mixed powder of 25% Na_2CO_3 and 75% K_2CO_3.

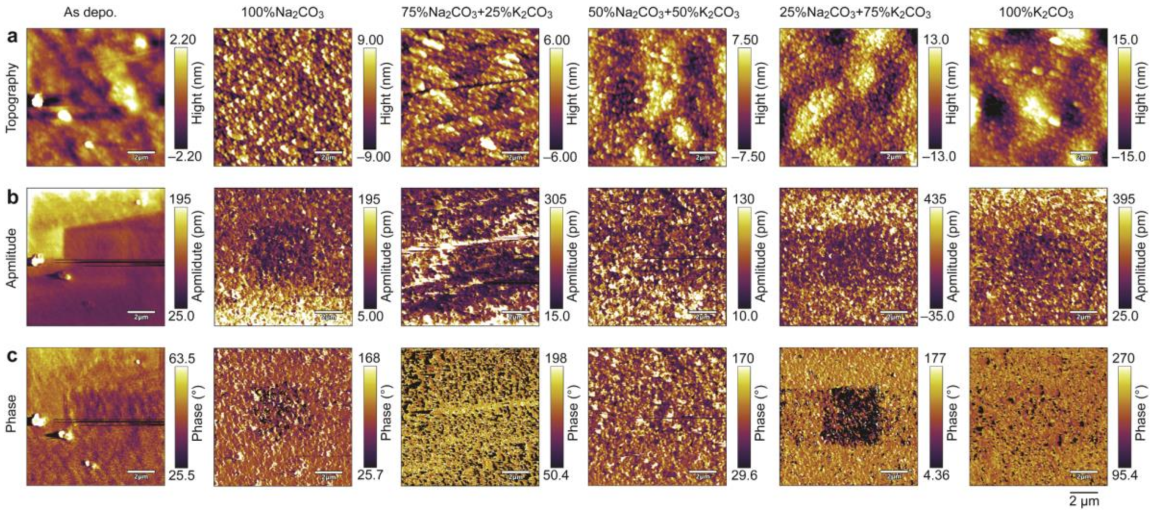

Figure S2. PFM images of the resultant films after applying switching bias of ±10 V. (a) Topography images. (b) Amplitude images. (c) Phase images.

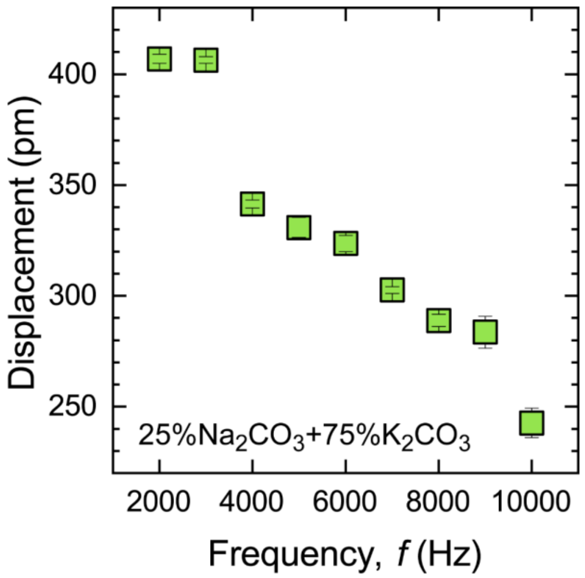

Figure S3. Frequency dependence of displacement of the film annealed in mixed powder of 25% Na_2CO_3 and 75% K_2CO_3.
